# Parasitic infections and resource economy of Danish Iron Age settlement through ancient DNA sequencing

**DOI:** 10.1371/journal.pone.0197399

**Published:** 2018-06-20

**Authors:** Katrine Wegener Tams, Martin Jensen Søe, Inga Merkyte, Frederik Valeur Seersholm, Peter Steen Henriksen, Susanne Klingenberg, Eske Willerslev, Kurt H. Kjær, Anders Johannes Hansen, Christian Moliin Outzen Kapel

**Affiliations:** 1 Department of Plant and Environmental Sciences, University of Copenhagen, Frederiksberg C, Denmark; 2 Centre for GeoGenetics, Natural History Museum of Denmark, University of Copenhagen, Copenhagen K, Denmark; 3 The Saxo Institute, University of Copenhagen, Copenhagen S, Denmark; 4 Trace and Environmental DNA (TrEnD) Laboratory, Department of Environment and Agriculture, Curtin University, Perth, Western Australia, Australia; 5 Environmental Archaeology and Material Science, National Museum of Denmark, Kongens Lyngby, Denmark; 6 Ancient Cultures of Denmark and the Mediterranean, National Museum of Denmark, Kongens Lyngby, Denmark; 7 Department of Zoology, University of Cambridge, Cambridge, United Kingdom; 8 Sanger Institute, Hinxton, Cambridge, United Kingdom; Seoul National University College of Medicine, REPUBLIC OF KOREA

## Abstract

In this study, we screen archaeological soil samples by microscopy and analyse the samples by next generation sequencing to obtain results with parasites at species level and untargeted findings of plant and animal DNA. Three separate sediment layers of an ancient man-made pond in Hoby, Denmark, ranging from 100 BC to 200 AD, were analysed by microscopy for presence of intestinal worm eggs and DNA analysis were performed to identify intestinal worms and dietary components. Ancient DNA of parasites, domestic animals and edible plants revealed a change in use of the pond over time reflecting the household practice in the adjacent Iron Age settlement. The most abundant parasite found belonged to the *Ascaris* genus, which was not possible to type at species level. For all sediment layers the presence of eggs of the human whipworm *Trichuris trichiura* and the beef tapeworm *Taenia saginata* suggests continuous disposal of human faeces in the pond. Moreover, the continuous findings of *T*. *saginata* further imply beef consumption and may suggest that cattle were living in the immediate surrounding of the site throughout the period. Findings of additional host-specific parasites suggest fluctuating presence of other domestic animals over time: *Trichuris suis* (pig), *Parascaris univalens* (horse), *Taenia hydatigena* (dog and sheep). Likewise, alternating occurrence of aDNA of edible plants may suggest changes in agricultural practices. Moreover, the composition of aDNA of parasites, plants and vertebrates suggests a significant change in the use of the ancient pond over a period of three centuries.

## Introduction

Paleoparasitology investigates ancient parasites found in soil, deposits, coprolites or mummified bodies. Particularly intestinal helminths are suited for paleoparasitological studies as the wall of their eggs has developed to resist alternating exposures to microbial, chemical or physical conditions when they are naturally excreted to the external environment [1]. By classical microscopy, Ferreira et al. found *Trichuris* eggs in 30,000 years old animal coprolites [2], and a range of helminth eggs have been identified in archaeological samples of different age and from many different global locations[3, 4, 5]. Likewise, in archaeological samples of Roman age from Europe and the Mediterranean it is evident that two genera are particularly prevalent, *Trichuris* spp. *and Ascaris* spp. [6]. Moreover, eggs of *Ascaris lumbricoides*, *Trichuris trichiura*, *Fasciola* spp., and *Diphyllobothrium* spp. have been demonstrated in samples from Germany and the Netherlands (ranging from 150 BC to 500 AD) [3]. A bog mummy from Denmark, the so-called Tollund man found in 1950 (dated to 375–210 BC [7]) was found to have been infected with *T*. *trichiura* [8].

Additionally, the presence of a parasite egg with a particular lifecycle may provide information on hygiene, resource economy and diet of past populations. High prevalence of soil-borne helminths that infect through direct faecal-oral transmission (e.g. *Ascaris* spp. and *Trichuris* ssp.) may suggest a low hygienic level with less confined faecal deposition. Furthermore, the presence of eggs from helminths that require a specific intermediate host for transmission (e.g. cattle, pigs, dogs) suggests the presence of such host in the immediate vicinity to the humans (or the ancient habitation); the presence of beef tapeworm (*Taenia saginata*) is consistent with rearing and consumption of undercooked beef and the giant tapeworm (*Diphyllobothrium latum*) with consumption of salmonid fish [9, 10, 11].

In general, morphological examination only allows for identification at the genus level, which prevents a conclusive assignment of a particular parasite species to a human or animal host. This is exemplified by the uniform egg morphology within the whipworm genus *Trichuris* and the tapeworm genus *Taenia*[12,13]. Thus, assignment of a recovered helminth egg at the species level has previously been based on the source of sampling, e.g. human coprolites, latrine sediment, samples taken directly from a mummy or the pelvic areas of a buried person, as the recovered egg could otherwise originate from animals [6]. As a complementary approach, isolation and typing of the helminth DNA provides a powerful tool for identification of parasites at the species level and can be used to elucidate the past transmission biology.

Although many microorganisms may be rapidly degraded over time, the hard shell of many soil transmitted helminths provides protection from the surrounding soil and allows a stable development from undifferentiated cell mass into larvae. Provided that the soil is moist, the eggs may exhibit extraordinary stress tolerance and may remain infective for months [14] or even years [15] after being released from the previous host. The stability of the eggs as an adaptation to soil transmission implies that the DNA in the egg is relatively well protected and can be isolated from the soil matrix, making eggs more ideal as markers for molecular methods. DNA based paleoparasitology has traditionally been limited to PCR amplicons from first-generation sequencing [9, 10, 11]. Recently, Søe et al. and Seersholm et al. have used shotgun sequencing to analyse parasite eggs extracted from soil samples [16, 17].

In the present study, we examine extracted soil samples from different fill-layers in a man-made pond excavated at Hoby in Denmark and dated to the Iron Age. The samples were screened for presence of parasite eggs by morphological examination and reads based on shotgun sequencing were analysed. Sequencing reads originating from aDNA were uniquely assigned to parasites, plants and vertebrates. Identification of helminths with distinct lifecycles as well as vertebrates and plants provides important insights into the resource economy of the adjacent settlement. However, morphological studies of plants and vertebrates were not made in this study.

## Material and methods

### The archaeological site at Hoby, Denmark

The Iron Age settlement and associated activities have been demonstrated over an area of 150 x 100 m, and around one third of this has been excavated. Across large parts of the settlement area the culture layer is preserved up to a thickness of 60–65 cm. The remains of 52 buildings have been demonstrated—large longhouses, small longhouses and other minor buildings. Further to these are wells, pits, cooking pits, two large man-made ponds and massive refuse accumulations containing bones.

The Centre of the settlement is dominated by three long-houses, one of which is surrounded by a fence. The three buildings stand out from the others on account of their overwhelming dimensions, their construction and the long period of their use. Usually common houses at that time had a stable at one end. The large buildings in Hoby differ by being used exclusively for residential purposes.

The valuable drinking set in the Hoby grave has been interpreted as a reward for participation in a punitive expedition or a diplomatic gift. Both interpretations would have required the Hoby magnate to control a larger area. The various building types and finds indicate that gatherings must have taken place at the site. The numerous cooking pits, the man-made pond holes and the offerings that were made in them, and perhaps the areas with huge deposits of bones, should be interpreted as evidence of gatherings involving many people from the various areas which the Hoby magnate controlled or was in alliances with. [18]

**Fig 1:**
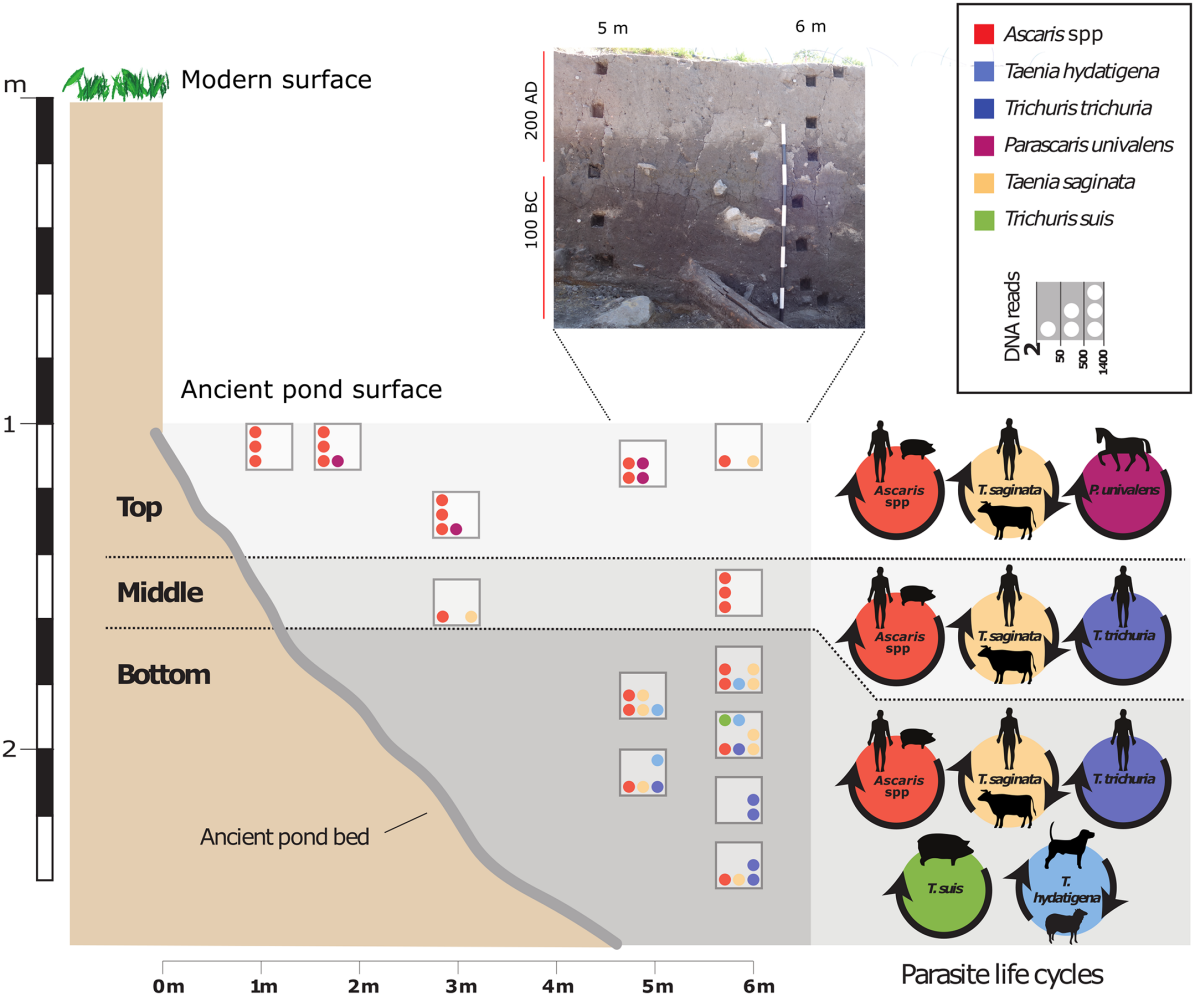
Excavation of fill layers in an Iron Age man-made pond in Hoby, Island of Lolland, Denmark. The image shows the face of the cross section of the pond with square holes after sampling, the red bars represents ^14^C dating supplemented with typological dating of artefacts. The graphic shows corresponding position (depth and distance from the edge) of samples (square boxes and sample ID). The numbered boxes show sample number and indicate the approximate position of sampling while circles inside reflect the number of reads assigned to specific parasites (see legend box top right). Bottom right: typical lifecycles of the parasites in the three layers based on the DNA assignments.

An artificial man-made pond dating to the Iron Age (100 BC -200 AD) was excavated in 2015 at Hoby (Fig 1); its top layers were revealed approximately one meter below the ground surface. The pond, an oval structure measuring roughly 15x18 m, was situated just 20 m north of the contemporary settlement. Hoby is a well-known archaeological location on the island of Lolland that has produced one the most exclusive male burials in Northern Europe, containing a number of Roman imports [19]. The pond was evidently filled with household refuse such as animal bones (mainly of domestic livestock), pottery, wood and stones. The base of the pond was subdivided into sections by wattled fences. Large trunks of wood were discovered at the bottom, thus leaving the intended function of the pond open for speculation. Plant macrofossils collected from the settlement area and the pond suggest that people who lived in the adjacent village were farming hulled barley (*Hordeum vulgare subsp*. *vulgare*), bread wheat (*Triticum aestivum*), rye (*Secale cereale*), oats (*Avena sativa*) and flax (*Linum usitatissimum*). The discovered animal bones suggest rearing of pigs, cattle, sheep and horses. Around the pond several cooking pits and two water wells were located, indicating food preparation at the pond. Sediment samples collected for investigation of parasite eggs and DNA analysis were extracted from an east-west cross section measuring 15 m, in 5 regularly spaced series from the edge towards the centre (Fig 1). Three distinct fill layers were readily observed. The basal sediments consist of clayey gyttja with a loss-on-ignition estimated organic content of 20–25% and a large amount of bone and clastic refuse material. The middle sediment clay layer has an organic content of c. 5% with a large amount of charcoal and occasional bone and clastic material. Both layers were deposited in standing water and the high content of clay particles were most likely washed into the pond from its immediate surroundings. The uppermost layer consisted of a poorly-sorted, sandy deposit as per the cultural sediments that cover the whole site. The pond was already filled with sediment prior to the deposition of this layer. The basal and the middle layer were dated by Accelerator Mass Spectrometry (AMS) to 70 BC (+/-45) and 97 AD (+/-27 AD), respectively. The topmost layers were dated according to the existing pottery typology to a period around 200 AD.

Complementary, C14 dating was carried out on three samples from the centrally located, thus deepest, vertical sequence: 1), the bottom of the pond, 157–158 cm top down, dates the period of creation of the pond to between BC 165 and AD 20; 2) 113–114 cm top down of the pond dates the top of the lowest layer to AD 30–40 and AD 50–135; 3) 99–100 cm top down of the pond is attributed to the middle layer: AD 55 to 135 (See S1 Table).

Additionally a diatom analysis was done (See S9 Table).

### Egg extraction and DNA sequencing

Initially, 19 sediment samples were screened for presence of intestinal parasite eggs (only *Ascaris* spp., *Trichuris* spp. and *Taenia* spp. eggs were found) in order to identify samples with evidence of human presence. Samples (5 gram) were dissolved, floated in high salt/sugar buffer and wet sieved through nylon filters (range first 100 μm then 22.4 μ m). The filtered section of samples were examined by microscopy after Søe et al. [11], and the remaining sediment of the sample was discarded. Thirteen of the samples were selected for further processing, from which 192 g (16 x 12 g) per sample were processed for egg isolation as described previously [11], and the remaining sediment of the sample was discarded. Of the filtered samples, 10% was subjected to morphological examination (see findings in S2 Table) and 90% for subsequent DNA analyses. The egg extraction was done in a lab designated to ancient soil samples at the Department of Plant and Environmental Sciences, University of Copenhagen and two extraction controls were done to detect any contamination.

Filtered samples (0.01 g– 0.47 g, average of 0.07 g, see S2 Table) were processed for DNA extraction and blunt end DNA library preparation in a dedicated aDNA laboratory at the Centre for GeoGenetics (CGG), Natural History Museum, University of Copenhagen, as previously described [11, 16]. Libraries were pooled at equimolar concentrations of 15 nM before shotgun sequencing using 80-bp single read chemistry on a HiSeq 2000/2500 platform at The Danish National High-Throughput DNA Sequencing Centre.

### Negative controls

Negative controls were prepared to detect contamination at all the relevant steps. Two negative controls initiated with egg extraction (by adding flotation buffer to an empty falcon tube), one with library preparation (by adding a tube with only buffer and enzyme) and one with PCR amplification (by adding a tube with only buffer and enzyme). From the initiation of the negative control to the finished analysed data, the samples and controls were treated the same.

Control samples of the modern surface and sediments were not collected.

### Sequence analysis

Sequencing data was pre-processed to remove adapter sequences, low-quality reads and reads shorter than 30 nt as previously described [16], the only exception being at the SGA—String Graph Assembler preprocess command step, option dust-threshold was set to 4. Next, helminth, vertebrate and plant DNA was identified based on alignment to mitochondrial and plastid genomes databases from NCBI using the ‘lowest common ancestor’ (LCA) approach as previously described[17]. Reads mapping to helminths (reads assigned within the phyla ‘platyhelminthes’ and ‘nematoda’) with 2 or more reads assigned to species level are reported (Fig 1), the only exception being 7 reads assigned to *Taenia asiatica*. These reads were excluded as they are assumed to stem from degraded *T*. *saginata* DNA. This is supported by the fact that 5 of the 7 reads show a single nucleotide mismatch and the remaining 2 reads showed 2 mismatches compared to *T*. *saginata* (see S3 Table). Reads assigned to *Ascaris*, *Ascaris suum* and *Ascaris lumbricoides* have been collapsed to the genus level *Ascaris* due to the close relation between *A*. *suum* and *A*. *lumbricoides*.

### Authenticity of sequences

**Fig 2:**
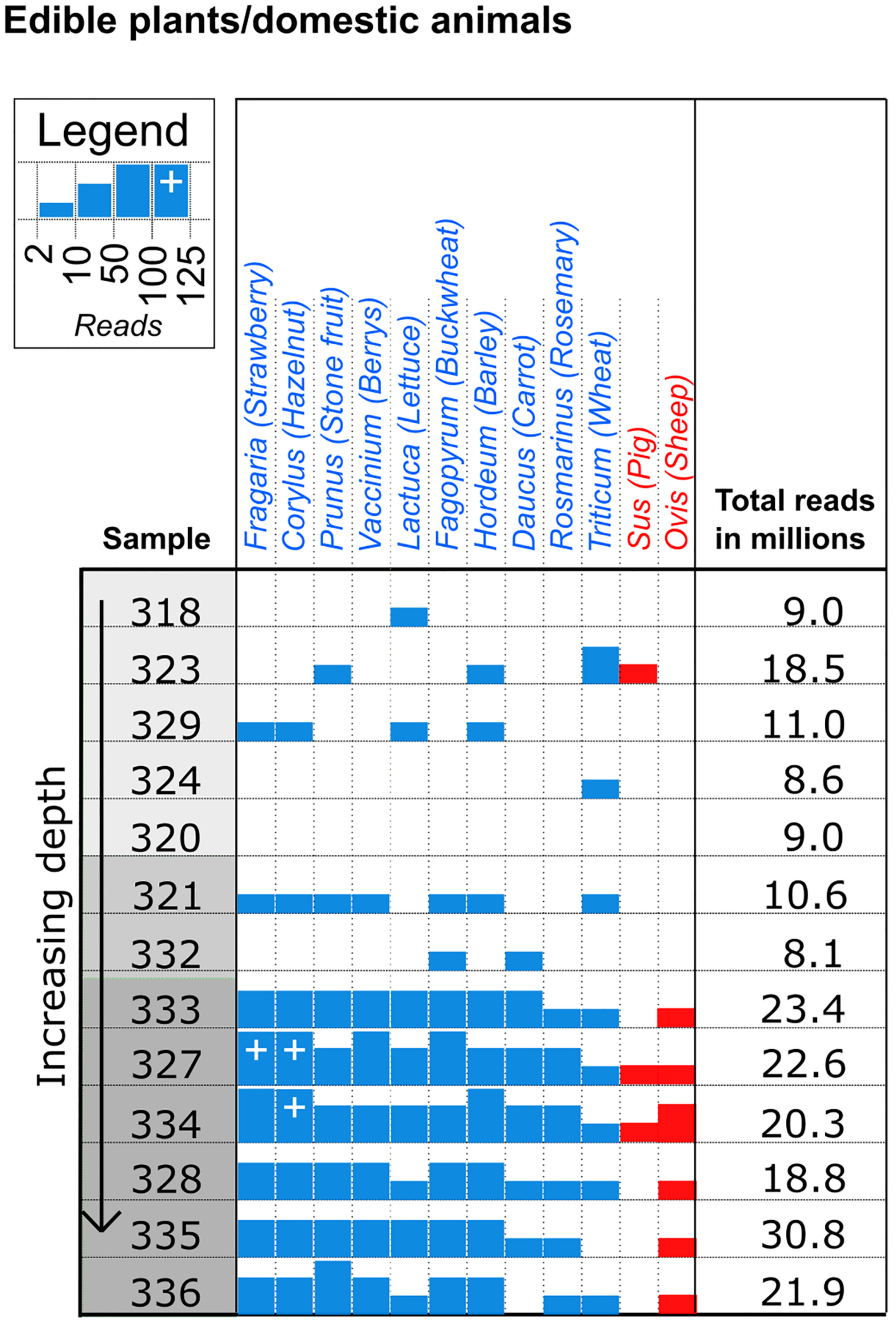
Edible plants and domestic animals detected in the samples. The most abundant edible plants (blue), in decreasing order left to right, and the two identified domestic animals (red) are shown. Samples from the top, middle and bottom layers are shaded in light grey, grey and dark grey, respectively. The total number of reads sequenced is presented per million reads (See S2 Table for exact numbers).

Reads assigned to vertebrates with more than one unique hit are reported at genus level (Fig 2). Number of reads assigned has been collapsed to the genera *Ovis* and *Sus* (See S4 Table). The following taxa have been excluded from the reported vertebrate results: *Homo sapiens* and related primates, *Meleagris gallopavo* (Wild Turkey), *Bos* spp. (Cattle), *Gallus* spp. (Junglefowl) since they are common laboratory contaminants [16][20] and were observed in both egg extraction controls. Furthermore, *Tupaia belangeri* (northern tree shrew native in Asia), *Calotes versicolor* (oriental garden lizard), *Pelodiscus sinesis* (Chinese Softshell Turtle), *Rana dybowskii* (Dybowski’s frog native in Asia) which were identified in a couple of samples were excluded. As these species are exotic to Denmark and were identified with a maximum of 3 reads, these identifications are considered to be misassignments.

Plastid sequence assignations are reported at the genus level, as the database in its current form is lacking many species (See S4 Table). The 10 most abundant genera are reported (Fig 2). Genera with 1 or more read assignations from controls are not reported. Full lists of helminth, vertebrate and plastids hits are presented in S5, S6 and S7 Tables.

### Genome analysis

*Ascaris* spp. assigned reads were extracted and remapped as previously described[16]. All *Ascaris* assigned reads were remapped against the KC839986 reference sequence, coverage and depths were calculated using Paleomix version 1.1.1, commands coverage and depths [21] (see S1 Fig).

Mitochondrial consensus sequences were called for *Ascaris* sequences using the commands samtools mpileup, bcftools call (version 1.2) and vcfutils vcf2fq. The consensus sequences were aligned with all mitochondrial genome sequences of the *Ascaris* genus publicly available at NCBI. Certain reference sequences were re-arranged to start with the cox1 gene. Alignment was performed with mafft v7[22], using the globalpair setting at 1000 iterations. The consensus sequence was uploaded to https://www.ncbi.nlm.nih.gov/ with accession number MH059555.

Genome analysis was not performed on DNA reads mapping to other parasites, as the number of hits were far from sufficient to generate a complete consensus sequence of their respective mitochondrial genomes.

To exclude contamination with modern DNA, ancient DNA damage patterns were assessed using mapDamage v2[23] as reported for *Ascaris* assigned reads in sample 323, as well as *Parascaris univalens* in sample 334, *Taenia saginata* in sample 334 and hazelnut (*Corylus*) in sample 327 (see S2 Fig). The same was done for spinach (*Spinacia*) reads from sample 329 to show modern DNA profile. In addition, the 5’ C to T transition percentage and the 3’ G to A transition percentages were also calculated and their averages are reported for embryophyta (land plants) and helminths in (S4 Fig).

## Results

Excavation of an ancient man-made pond in Hoby, Denmark, identified three fill layers of varying physical properties recognised visually through different coloration (200 BC– 100 AD). Representative sediment samples were carefully extracted and processed for microscopic identification and ancient DNA analyses with focus on parasitic, plant and vertebrate remains (Fig 1).

*Ascaris* spp. eggs were identified in all sediment samples although more abundant in the top and middle layers. Eggs from *Taenia* spp. and *Trichuris* spp. were recovered primarily from the bottom layer, but few eggs (1 *Taenia* spp. and 2 *Trichuris* spp. in the 10% sample subset) were detected in the middle layer. DNA shotgun sequencing of the samples resulted in read assignments for *Trichuris* being almost unambiguously assigned to the human *T*. *trichiura*, apart from 2 reads assigned to *T*. *suis* (bottom layer). Similarly for *Taenia*, the majority of sequencing reads were assigned to *T*. *saginata* (samples from all layers) and few to *T*. *hydatigena* (bottom layer only). DNA analyses also identified *Parascaris univalens*, although not observed during initial microscopy on a subsample. *Ascaris* spp. was the most abundant parasite finding based on sequencing reads (all layers).

The damage patterns of the *Ascaris* spp. DNA (S2 Fig) authenticates the ancient nature of the DNA from sample 323, by identifying increasing C to T (19%) and G to A (12%) transitions at the respective 5’ and 3’ ends of assigned sequencing reads. Reads assigned to *Parascaris univalens* (sample 334, 315 reads) and *Taenia saginata* (sample 334, 151 reads) also display aDNA damage patterns (S2 Fig) although less pronounced. In addition, a mitochondrial genome of ancient *Ascaris* was reconstructed from the top layer of the pond (sample 323) with 96% coverage. The only parts without coverage were the repetitive regions at bp 7222–7258, 7448–7604 and 7670–7911, which were removed in process of filtering reads of little information (see S1 Fig).

The genera and distribution of identified edible plants and domestic animals show different compositions between the layers (Fig 2). Rosemary (*Rosmarinus*) was only detected in the bottom layer. Strawberry (*Fragaria*), hazelnut (*Corylus*), stone fruits (*Prunus*), berries (*Vaccinium*), lettuce (*Lactuca*), buckwheat (*Fagopyrum*), barley (*Hordeum*), carrot (*Daucus*) and wheat (*Triticum*) were found in all three layers but predominantly in the bottom layer. Sheep (*Ovis*) was only detected in the bottom layer and pig (*Sus*) in the top layer, and in higher abundance in the bottom layer. It was possible to generate an aDNA damage profile for Hazelnut (*Corylus*) from the 107 reads in sample 327.

Spinach (*Spinacia*) was initially detected in all samples, but subsequently excluded as the DNA damage profile was not consistent with ancient DNA (in sample 329, 146 reads).

Notably, ancient DNA damage profiles as indicated for embryophyta (land plants) and helminths have different patterns (S4 Fig). Average DNA damage of the first 5’ and 3’ end position is decreasing with increasing depth for helminth assigned reads (20% → 5%), this is due to a decreasing number of reads with increasing depth, while only the bottom layer exhibits ancient DNA damage profiles for reads assigned to plants. A DNA damage below 5% is observed in the plant DNA recovered from the top layer (S4 Fig). In the middle and bottom layer, the DNA damage increases to 5–10%. From two samples (321 and 335) the helminth data were excluded from the figure due to very low number of assigned reads (S7 and S8 Tables).

## Discussion

In the three layers of the pond, the different compositions of parasite-, plant- and vertebrate aDNA reflect a varying deposition patterns over time (Fig 1). Due to the initial sectioning of the man-made pond by wattled fences, several hypotheses have been proposed for its purpose: a ritual structure, a fishpond, a structure for maturing wood for instance for waggon building. However, this study examines the sediment of the fill layers in the pond and thereby focuses on the resource economy and lifestyle of the humans’ inhabitation the site therefore what the pond was filled with after its intended original use. In particular, the identification of parasites which are only excreted with human faeces, *i*.*e*. *Trichuris trichiura* and *Taenia saginata*, indicate that the pond was gradually filled with human faeces towards the beginning of the 1st century AD. This accumulation continued until the end of the 2^nd^ century AD, at which the distribution of diatoms indicates that the pond had dried out, with more moist conditions occurring only during rainy seasons, (S9 Table). This is probably because the pond at this stage had been filled with sediments above the groundwater level.

The identification of the beef tapeworm *Taenia saginata* across all three layers, suggests that the pond has been used for deposition of human faeces over the entire period. Detection of *P*. *univalens* only in the top layer and *Taenia hydatigena*, *Trichuris trichiura* and *Trichuris suis* in the bottom layer, suggests a change in the resource economy or disposal habits on the site.

The presence of eggs of the human whipworm *T*. *trichiura* in the bottom layer (100 BC-0) suggests that the pond was used for disposing human faeces, as it has a direct lifecycle in man with no need for intermediate hosts for transmission and requires maturation in the external environment. The top layer contains *P*. *univalens*, which suggests depositions of horse manure. Horses were used for transportation at the time and were extremely valued and therefore indicators of high status; such finding supports an increase in welfare in Hoby from 100 BC towards 200 AD.

The identification of the *Ascaris* spp. in all three layers suggests that either human faeces or pig manure has been deposited in the pond. The *Ascaris* genus includes several soil transmitted species where the eggs require maturation in the external environment [24]. The pig (*A*. *suum*) and the human (*A*. *lumbricoides*) roundworms are closely related [25], with the potential to cross-infect. They might even be considered conspecific [26], making it impossible to assign the origin of the infection to humans, pigs or both species.

The consistent presence of the beef tapeworm, *T*. *saginata*, in all layers suggests a continuous consumption of raw or undercooked beef throughout the period (100BC-100AD), which further indicate domestic cattle household in the vicinity of the site and that human defecate was deposited in the pond. *T*. *saginata* has not previously been detected using ancient DNA analyses in Viking or Medieval age samples from Northern Europe [16]. Unlike in Spain and Iran where they have been found in samples dating back to 7250–7050 BC and 2500–1500 BC, respectively [27]. The presence of *T*. *hydatigena*, in the bottom layer supports the presence of domestic sheep and dogs at the early Hoby settlement (100BC-0) and *T*. *suis* further indicates presence of pigs in this period. Both *T*. *hydatigena* and *T*. *suis* have been molecularly typed by Søe *et al*. in samples from Denmark dated to 1375-1680AD [16], with the present finding being approximately 1500 years older.

Consistent with previous dating of archaeological artefacts excavated from the ancient man-made pond in Hoby, and supplementary C14 dating, the majority of the DNA sequences from samples of the pond layers had a clear damage profile characteristic of ancient DNA. The only exception to this was low damage profiles of one species (spinach) from the top layer (S8 Table), which can be explained by modern plant roots penetrating the top layer or DNA leaching through the soil layers as described by Andersen *et*. *al*. [28].

Identification of plants in the pond, can result from kitchen waste, human faeces [16][17] or be a result of natural deposition from plants growing adjacent to the pond. The plant aDNA data (Fig 2) reveals a greater diversity and abundance of edible plants in the bottom layer. It is uncertain whether this is a real difference in the original composition or a result of different preservation conditions. The same pattern was seen in the recovery of plant macrofossils where many seeds and other plant remains were preserved in the oxygen free bottom layers in contrast to the top layers where only carbonised plant remains had survived. aDNA from wheat and barley correlates with the plant macro fossil findings from the pond. However, aDNA from strawberry, hazelnut, stone fruits (probably *Prunus avium* or *P*. *spinosa*) and carrot does not correlate with the macro fossil assemblage from Hoby. The identifications are on the other hand very plausible as these species are known from other Iron Age macro fossil finds from Denmark [29]. Together with the aDNA profile from hazelnut, this indicates the usefulness of aDNA analysis in the identification of plants in ancient samples. Carrots were probably wild plants that grew in the vicinity of the pond, whether these plants were collected as food plants is not possible to establish based on our findings. The identification of cranberry is likely, although these species are seldom seen on the fertile soils around Hoby. The detection of lettuce, buckwheat and rosemary is unexpected as these species are only known from the medieval period or later. Unfortunately there are too few reads of these species to get ancient DNA damage profiles, hence modern contamination cannot be ruled out. DNA from lettuce and buckwheat could stem from cultivation in modern times. The rosemary that was found in the bottom layer is more interesting as this species requires a warm climate and therefore it is very unlikely that it has been cultivated at Hoby. It is however possible that the rosemary has been imported as a dried spice in the Iron Age.

The higher abundance of pig (*Sus)* and sheep (*Ovis)* in the bottom layer may complement the observations done for the plants and support the assumption that kitchen waste or human faeces was deposited in the pond. Furthermore, these two vertebrates serve as hosts for the parasites *T*. *hydatigena* (sheep) and *T*. *suis* (pig) found in the same layer.

Overall, the results are consistent with a pastoralist based settlement as expected from the time period with primarily sheep and pigs round 100BC-0 and cattle rearing throughout the period (100 BC-200AD). From this time, pigs and cattle appear to be part of the domesticated animals at the settlement. Only around 200AD the horse appears which really supports a major shift in the resource economy of the settlement. In conclusion, the aDNA analyses show that through the period 100 BC- 200 AD the pond has been used for kitchen waste, human excrements and animal dung, with a change in diet/habits over time. This study demonstrates the enormous potential that the new methods, especially the studies of aDNA offer towards the understanding and interpretation of archaeological contexts.

## Supporting information

S1 Table Carbon 14 dating: ^14^C data obtained from the Hoby pond. Laboratory numbers are reported in column one. The interception of the radiocarbon age with the calibration curve is shown in column four.
(PDF)

S2 Table Sample information. Counts in column two to four represent the number of parasite eggs found in 10% filtered sample by microscopy; Column eight and nine show the number of reads assigned to mitochondrial and plastid DNA, respectively. Column ten show the number of hits (both mitochondrial and plastid hits) per million total reads.
(PDF)

S3 Table Collapsed reads assigned to genera. List of collapsed genera and what subdivisions they contain.
(PDF)

S4 Table Taenia asiatica mismatch identification, based on NCBI blast. The seven *Taenia asiatica* assigned reads were blasted and the results from *Teania asiatica* and *Taenia saginata* is shown to compare the mismatches from read to database genomic DNA.
(PDF)

S5 Table Helminth reads identified in samples. Show the number of reads assigned to named helminths. Sample number and negative controls, extraction blank 1(EX1), extraction blank 2 (EX2) library preparation blank (LIB blank) and PCR preparation blank (PCR blank) in top row.
(PDF)

S6 Table Plant reads identified in samples. Show the number of reads assigned to named plants. Sample number and negative controls; extraction blank 1(EX1), extraction blank 2 (EX2) library preparation blank (LIB blank) and PCR preparation blank (PCR blank) in top row.
(PDF)

S7 Table Vertebrate reads identified in samples. Show the number of reads assigned to named vertebrates. Sample number and negative controls; extraction blank 1(EX1), extraction blank 2 (EX2) library preparation blank (LIB blank) and PCR preparation blank (PCR blank) in top row.
(PDF)

S8 Table Ancient DNA damage profiles. Shows the MapDamage percentage calculated for land plants (*Embryophyta*) and platyhelminthes and nematodes (Helminth). 3’ G>A % represents the percentage of guanine nucleotides which has been degraded to adenine nucleotides at the 3’ end of the read. 5’ C>T % represents the percentage of cytosine nucleotides which has been degraded to thymine nucleotides at the 5’ end of the read.
(PDF)

S9 Table Analysis of diatoms, phytoliths & ash pseudomorphs in soil samples. Amount of phytoliths, diatoms and ash pseudomorphs in sediment samples collected from the central part (at 6 m from the Eastern edge) of the artificial dam in Hoby and their calculated concentrations (per g). Sample weight indicates the portion of the homogenized sediment used for the extraction procedures. The sample is mixed with 50 μl of 6N HCl and 450 μl SPT (3Na2WO4·9WO3·H2O), and a representative aliquot of 50 μl of the supernatant is removed and placed on a microscope slide for counting. Phytoliths and diatoms are counted in 10 evenly distributed fields on a microscope slide at 200x magnification, while ash pseudomorphs were counted in 15 fiels at 400x magnification. The area covered by each field depends on the magnification. 50 μl of the supernatant represent 10% of the total solution. Based on these numbers it is possible to calculate the concentration of phytoliths, diatoms and ash pseudomorphs in each sample.
(PDF)

S1 Fig Coverage and depth of the reconstructed Ascaris spp. genome. **A)** Coverage in percent of the mitochondrial genome reference sequence (KC839986) is shown (green) as well as the average depth of coverage (red). Samples from the top, middle and bottom horizons are shaded in light grey, grey and dark grey, respectively. **B)** Read coverage (blue) for *Ascaris* spp. reads from sediment sample 323 across the mitochondrial genome, red line shows average coverage (6.42x).
(TIF)

S2 Fig Ancient DNA damage profiles. Shows ancient DNA damage profile plots of *Ascaris* spp. (sample 323, C to T (19%) and G to A (12%))*Parascis univalens* (sample 334, C to T (8%) and G to A (6%)), *Taenia saginata* (sample 334, C to T (5%) and G to A (9%)) and hazelnut (*Corylus*) (sample 327, C to T (16%) and G to A (10%)). C to T (red) and G to A (blue) transitions substitutions for 5’ end of reads (1 to 25 on x-axis) and for 3’ end of reads (-25 to -1 on the x-axis).
(TIF)

S3 Fig Modern DNA profile of Spinach (*Spinacia*). Shows modern DNA damage profile plots of Spinach (*Spinacia*, sample 329) with no increased damage towards the ends of the reads. C to T (red) and G to A (blue) transitions substitutions for 5’ end of reads (1 to 25 on x-axis) and for 3’ end of reads (-25 to -1 on the x-axis).
(TIF)

S4 Fig Ancient DNA damage detected in plant and helminth reads. The figure shows the collective ancient DNA damage (C to T and G to A transitions in the 5’and 3’respective ends of assigned reads) as percentage of the 13 samples listed by increasing depth with the top layer (light grey) middle layer (grey) and the bottom layer (dark grey).
(TIF)

S1 File Extraction & counting of phytoliths, diatoms, sponges, radiolarians and charred organic material. (DOCX)
