# Supplementary material for: Parasitic infections and resource economy of Danish Iron Age settlement through ancient DNA sequencing
Source: PLoS One. 2018 Jun 20;13(6):e0197399. doi: 10.1371/journal.pone.0197399 (PMC6010210; doi:10.1371/journal.pone.0197399)
Supplement: S1 Table — Laboratory numbers are reported in column one. The interception of the radiocarbon age with the calibration curve is shown in column four. (PDF) [file pone.0197399.s001.pdf]

Laboratory number	***Conventional radiocarbon age***	***Calibrated Result***	***Intercept of radiocarbon age with calibration curve***
Beta-436806 , 157-158 cm from top of the pond	2050 +/- 30 BP	BC 165 to AD 20 (Cal BP 2115 to 1930)	Cal BC 45 (Cal BP 1995)
Beta-436807 , 113-114 from the top of the pond	1910 +/- 30 BP	Cal AD 30 to 40<br>(Cal BP 1920 to 1910)<br>and<br>Cal AD 50 to 135<br>(Cal BP 1900 to 1815)	Cal AD 80 (Cal BP 1870)
Beta-436808 , 99-100 cm from the top of the pond	1900 +/- 30 BP	Cal AD 55 to 135 (Cal BP 1895 to 1815)	Cal AD 85 (Cal BP 1865)
