# Supplementary material for: Parasitic infections and resource economy of Danish Iron Age settlement through ancient DNA sequencing
Source: PLoS One. 2018 Jun 20;13(6):e0197399. doi: 10.1371/journal.pone.0197399 (PMC6010210; doi:10.1371/journal.pone.0197399)
Supplement: S2 Table — Counts in column two to four represent the number of parasite eggs found in 10% filtered sample by microscopy; Column eight and nine show the number of reads assigned to mitochondrial and plastid DNA, respectively. Column ten show the number of hits (both mitochondrial and plastid hits) per million total reads. (PDF) [file pone.0197399.s002.pdf]

Sample no. *Top* *Middle* *Bottom*	Trichuris *spp.*	Ascaris *spp.*	Taenia *spp.*	*Sample weight (g)*	*Total no. of reads*	*Duplicates discarded by pre-processing raw data*	*Mitochondrial hits*	*Plastid hits*	*No. of hits per million reads*
#318	-	3	-	0.02	9031423	5224816	2863	2570	602
#323	-	1	-	0.03	18546517	2100954	4937	2002	374
#329	1	-	-	0.01	11001676	4767637	3472	4040	683
#324	1	1	-	0.02	8643198	7841602	39314	9395	5636
#320	-	1	-	0.02	8984866	1983716	5803	3488	1034
#321	6	-	-	0.09	10568530	2690613	16197	18059	3241
#332	4	-	-	0.17	8143376	973939	6929	8707	1920
#333	-	1	-	0.01	23402404	1830413	4145	3376	321
#327	-	2	-	0.03	22561070	1676490	4464	2729	319
#334	4	3	2	0.06	20311913	3059988	11675	13477	1238
#328	1	2	1	0.05	18777413	1592324	15183	16206	1672
#335	5	-	1	0.47	30753335	1279463	10547	13499	782
#336	5	-	-	0.14	21899674	788291	8033	10634	852
EX1	-	-	-	0.02	357684	48486	262	173	1216
EX2	-	-	-	0.04	365474	104126	191	120	851
LIB blank	-	-	-	-	169070	21975	91	61	899
PCR blank	-	-	-	-	2690	22	1	2	1115
