# Supplementary material for: Parasitic infections and resource economy of Danish Iron Age settlement through ancient DNA sequencing
Source: PLoS One. 2018 Jun 20;13(6):e0197399. doi: 10.1371/journal.pone.0197399 (PMC6010210; doi:10.1371/journal.pone.0197399)
Supplement: S3 Table — List of collapsed genera and what subdivisions they contain. (PDF) [file pone.0197399.s003.pdf]

Genera	*Genera contains reads assigned to:*
Ovis	*Ovis* spp, *Ovis orientalis*, *Ovis aries*, *Ovis vignei*
Sus	*Sus* spp, *Sus scrofa*, *Sus scrofa taivanus*, *Sus scrofa domesticus*
Fragaria	*Fragaria* spp, *Fragaria chiloensis, Fragaria iinumae, Fragaria mandshurica, Fragaria vesca subsp. bracteata and Fragaria vesca subsp. vesca*
Corylus	*Corylus* spp. and *Corylus heterophylla*
Mentha	*Mentha longifolia*
Rheum	*Rheum palpatum*
Prunus	*Prunus* spp, *Prunus maximowiczii, Prunus mume, Prunus padus* and *Prunus yedoensis*
Vaccinium	*Vaccinium macrocarpon*
Lactuca	*Lactuca sativa*
Fagopyrum	*Fagopyrum esculentum subsp. ancestrale* and *Fagopyrum tatarium*
Hordeum	*Hordeum* spp*, Hordeum jubatum* and *Hordeum vulgare subsp. vulgare*
Daucus	*Daucus carota*
Triticum	*Triticum monococcum*
Rosmarinus	*Rosmarinus officinalis*
