# Supplementary material for: Parasitic infections and resource economy of Danish Iron Age settlement through ancient DNA sequencing
Source: PLoS One. 2018 Jun 20;13(6):e0197399. doi: 10.1371/journal.pone.0197399 (PMC6010210; doi:10.1371/journal.pone.0197399)
Supplement: S4 Table — The seven Taenia asiatica assigned reads were blasted and the results from Teania asiatica and Taenia saginata is shown to compare the mismatches from read to database genomic DNA. (PDF) [file pone.0197399.s004.pdf]

<table>
<tr><th>Sample</th><th>Read mapped against</th><th>Mapping</th></tr>
<tr><td>333</td><td>Taenia asiatica</td><td>ATTTAGTTGTGACATCAGCTGATGTCATTCATTCGTTTTCTGTTCCATCTTTAAATTTAA
||||||||||||||||||||||||||||||||||||||||||||||||||||||||||||
ATTTAGTTGTGACATCAGCTGATGTCATTCATTCGTTTTCTGTTCCATCTTTAAATTTAA</td></tr>
<tr><td></td><td>Taenia saginata</td><td>ATTTAGTTGTGACATCAGCTGATGTCATTCATTCGTTTTCTGTTCCATCTTTAAATTTAA
|||||||||||||||||| |||||| ||||||||||||||||||||||||||||||||||
ATTTAGTTGTGACATCAGTTGATGTTATTCATTCGTTTTCTGTTCCATCTTTAAATTTAA</td></tr>
<tr><td>333</td><td>Taenia asiatica</td><td>TCCTTACCATTTAGTTGTGACATCAGCTGATGTCATTCATTCGTTTTCTATTC
|||||| |||||||||||||||||||||||||||||||||||||||||| |||
TCCTTATCATTTAGTTGTGACATCAGCTGATGTCATTCATTCGTTTTCTGTTC</td></tr>
<tr><td></td><td>Taenia saginata</td><td>TCCTTACCATTTAGTTGTGACATCAGCTGATGTCATTCATTCGTTTTCTATTC
|||||||||||||||||||||||||| |||||| ||||||||||||||| |||
TCCTTACCATTTAGTTGTGACATCAGTTGATGTTATTCATTCGTTTTCTGTTC</td></tr>
<tr><td>333</td><td>Taenia asiatica</td><td>TTAGTGTGTATCGCTGAGTTAATAAGTTATGTTATTCGTCCAATAGTG
||||||||||||||||||||||||||||||||| |||||||| |||||
TTAGTGTGTATCGCTGAGTTAATAAGTTATGTTGTTCGTCCAGTAGTG</td></tr>
<tr><td></td><td>Taenia saginata</td><td>TTAGTGTGTATCGCTGAGTTAATAAGTTATGTTATTCGTCCAATAGTG
||||||||||| |||||||||||||||||| ||||||||||| | |||
TTAGTGTGTATTGCTGAGTTAATAAGTTATATTATTCGTCCAGTGGTG</td></tr>
<tr><td>334</td><td>Taenia asiatica</td><td>TATGCGTATGGTTTATGGTACTCCTTACCATTTAGTTGTGACATCAGCTGATGTCATTCA
||||||||||||||||||||||||||| ||||||||||||||||||||||||||||||||
TATGCGTATGGTTTATGGTACTCCTTATCATTTAGTTGTGACATCAGCTGATGTCATTCA

TTCGTTCTCTGTTCCATCT
|||||| ||||||||||||
TTCGTTTTCTGTTCCATCT</td></tr>
</table>

	Taenia saginata	TATGCGTATGGTTTATGGTACTCCTTACCATTTAGTTGTGACATCAGCTGATGTCATTCA<br>||||||||||||||||||||||||||||||||||||||||||||||| |||||| ||||| TATGCGTATGGTTTATGGTACTCCTTACCATTTAGTTGTGACATCAGTTGATGTTATTCA<br><br>TTCGTTCTCTGTTCCATCT<br>|||||| |||||||||||| TTCGTTTTCTGTTCCATCT
334	Taenia asiatica	AGCCTATGCATATGGTTTATGGTACTCCTTACCATTTAGTTGTGACATCAGCTGATGTCA<br>||||||||| ||||||||||||||||||||| |||||||||||||||||||||||||||| AGCCTATGCGTATGGTTTATGGTACTCCTTATCATTTAGTTGTGACATCAGCTGATGTCA<br><br>TTCATTCGTTTTCTGTTCCAT<br>||||||||||||||||||||| TTCATTCGTTTTCTGTTCCAT
	Taenia saginata	AGCCTATGCATATGGTTTATGGTACTCCTTACCATTTAGTTGTGACATCAGCTGATGTCA<br>||||||||| |||||||||||||||||||||||||||||||||||||||||| |||||| | AGCCTATGCGTATGGTTTATGGTACTCCTTACCATTTAGTTGTGACATCAGTTGATGTTA<br><br>TTCATTCGTTTTCTGTTCCAT<br>||||||||||||||||||||| TTCATTCGTTTTCTGTTCCAT
334	Taenia asiatica	ATAAGCCTATGCGTATGGTTTATGGTACTCCTTACCATTTAGTTGTGACATCAGCTGATG<br>|||||||||||||||||||||||||||||||||| ||||||||||||||||||||||||| ATAAGCCTATGCGTATGGTTTATGGTACTCCTTATCATTTAGTTGTGACATCAGCTGATG<br><br>TCATTCATTCGTTTTCTG<br>|||||||||||||||||| TCATTCATTCGTTTTCTG
	Taenia saginata	ATAAGCCTATGCGTATGGTTTATGGTACTCCTTACCATTTAGTTGTGACATCAGCTGATG<br>|||||||||||||||||||||||||||||||||||||||||||||||||||||| |||||

<table>
<tr><td></td><td></td><td>ATAAGCCTATGCGTATGGTTTATGGTACTCCTTACCATTTAGTTGTGACATCAGTTGATG

TCATTCATTCGTTTTCTG
| ||||||||||||||||
TTATTCATTCGTTTTCTG</td></tr>
<tr><td rowspan="2">334</td><td>Taenia asiatica</td><td>TGGTACTCCTTACCATTTAGTTGTGACATCAGCTGATGTCATTCATTCGTTTTCTGTTCC
|||||||||||| |||||||||||||||||||||||||||||||||||||||||||||||
TGGTACTCCTTATCATTTAGTTGTGACATCAGCTGATGTCATTCATTCGTTTTCTGTTCC

ATCTTTAAATTTAAAGATGGA
|||||||||||||||||||||
ATCTTTAAATTTAAAGATGGA</td></tr>
<tr><td>Taenia saginata</td><td>TGGTACTCCTTACCATTTAGTTGTGACATCAGCTGATGTCATTCATTCGTTTTCTGTTCC
|||||||||||||||||||||||||||||||| |||||| ||||||||||||||||||||
TGGTACTCCTTACCATTTAGTTGTGACATCAGTTGATGTTATTCATTCGTTTTCTGTTCC

ATCTTTAAATTTAAAGATGGA
|||||||||||||||||||||
ATCTTTAAATTTAAAGATGGA</td></tr>
</table>
