# Supplementary material for: Parasitic infections and resource economy of Danish Iron Age settlement through ancient DNA sequencing
Source: PLoS One. 2018 Jun 20;13(6):e0197399. doi: 10.1371/journal.pone.0197399 (PMC6010210; doi:10.1371/journal.pone.0197399)
Supplement: S5 Table — Show the number of reads assigned to named helminths. Sample number and negative controls, extraction blank 1(EX1), extraction blank 2 (EX2) library preparation blank (LIB blank) and PCR preparation blank (PCR blank) in top row. (PDF) [file pone.0197399.s005.pdf]

	#318	#320	#321	#323	#324	#327	#328	#329	#332	#333	#334	#335	#336	EX1	EX2	LIB blank	PCR blank
Ascaris	549	344	18	798	176	89	12	263	549	73	78	1	21	1	0	0	0
Ascaris_lumbricoides	50	25	1	58	21	9	1	18	35	5	3	0	3	0	0	0	0
Ascaris_suum	251	395	2	511	42	13	13	36	266	50	86	0	9	0	0	0	0
Parascaris_univalens	0	3	0	29	315	0	0	0	0	0	0	0	0	0	0	0	0
Taenia	0	0	1	0	0	7	2	0	0	11	18	0	0	0	0	0	0
Taenia_hydatigena	0	0	0	0	0	5	2	0	0	44	27	0	0	0	0	0	0
Taenia_saginata	0	0	6	0	0	80	5	2	0	108	151	1	3	0	0	0	0
Trichuris_suis	0	0	0	0	0	0	0	0	0	0	2	1	0	0	0	0	0
Trichuris_trichiura	0	0	0	0	0	0	40	0	1	0	2	68	78	0	0	0	0
