# Supplementary material for: Parasitic infections and resource economy of Danish Iron Age settlement through ancient DNA sequencing
Source: PLoS One. 2018 Jun 20;13(6):e0197399. doi: 10.1371/journal.pone.0197399 (PMC6010210; doi:10.1371/journal.pone.0197399)
Supplement: S6 Table — Show the number of reads assigned to named plants. Sample number and negative controls; extraction blank 1(EX1), extraction blank 2 (EX2) library preparation blank (LIB blank) and PCR preparation blank (PCR blank) in top row. (PDF) [file pone.0197399.s006.pdf]

	#318	*#320*	*#321*	*#323*	*#324*	*#327*	*#328*	*#329*	*#332*	*#333*	*#334*	*#335*	*#336*	*EX1*	*EX2*	*LIB blank*	*PCR blank*
Fragaria	0	0	8	0	0	114	20	2	1	22	47	32	18	0	0	0	0
Corylus	0	0	4	0	0	107	14	2	0	25	99	27	27	0	0	0	0
Prunus	1	0	3	1	0	41	9	0	0	17	12	12	64	0	0	0	0
Vaccinium_macrocarpon	1	0	2	1	0	52	12	0	0	44	44	21	14	0	0	0	0
Lactuca_sativa	2	0	0	0	0	42	4	3	1	17	26	11	5	0	0	0	0
Fagopyrum	0	0	3	0	0	40	12	0	2	38	26	14	12	0	0	0	0
Hordeum_vulgare_subsp._vulgare	0	0	1	2	0	23	8	1	0	15	32	31	14	0	0	0	0
Hordeum	0	0	1	0	1	23	4	2	0	12	24	16	8	0	0	0	0
Daucus_carota	0	0	0	0	0	13	6	1	3	11	20	5	0	0	0	0	0
Triticum_monococcum	1	0	2	15	4	5	3	1	1	9	6	0	2	0	0	0	0
Rosmarinus_officinalis	0	0	0	0	0	14	3	0	0	4	11	5	6	0	0	0	0
