# Supplementary material for: Parasitic infections and resource economy of Danish Iron Age settlement through ancient DNA sequencing
Source: PLoS One. 2018 Jun 20;13(6):e0197399. doi: 10.1371/journal.pone.0197399 (PMC6010210; doi:10.1371/journal.pone.0197399)
Supplement: S7 Table — Show the number of reads assigned to named vertebrates. Sample number and negative controls; extraction blank 1(EX1), extraction blank 2 (EX2) library preparation blank (LIB blank) and PCR preparation blank (PCR blank) in top row. (PDF) [file pone.0197399.s007.pdf]

	#318	#320	#321	#323	#324	#327	#328	#329	#332	#333	#334	#335	#336	EX1	EX2	LIB blank	PCR blank
Ovis	0	0	1	0	0	3	5	1	0	7	12	6	5	0	0	0	0
Sus_scrofa	1	0	0	0	0	4	1	0	0	0	1	1	0	0	0	0	0
Sus_scrofa_taivanus	0	0	0	2	0	0	0	0	0	0	0	0	0	0	0	0	0
Ovis_aries	0	0	0	0	0	1	0	0	0	0	0	0	0	0	0	0	0
Ovis_orientalis	0	0	0	0	0	1	0	0	0	0	0	0	0	0	0	0	0
Ovis_vignei	1	0	0	0	0	0	0	0	0	0	0	0	0	0	0	0	0
Sus	0	0	0	1	0	0	0	0	0	0	1	0	0	0	0	0	0
Sus_scrofa_domesticus	0	0	0	0	0	0	0	0	0	0	0	0	1	0	0	0	0
