# Supplementary material for: Parasitic infections and resource economy of Danish Iron Age settlement through ancient DNA sequencing
Source: PLoS One. 2018 Jun 20;13(6):e0197399. doi: 10.1371/journal.pone.0197399 (PMC6010210; doi:10.1371/journal.pone.0197399)
Supplement: S8 Table — Shows the MapDamage percentage calculated for land plants (Embryophyta) and platyhelminthes and nematodes (Helminth). 3’ G>A % represents the percentage of guanine nucleotides which has been degraded to adenine nucleotides at the 3’ end of the read. 5’ C>T % represents the percentage of cytosine nucleotides which has been degraded to thymine nucleotides at the 5’ end of the read. (PDF) [file pone.0197399.s008.pdf]

	Embryophyta			*Helminth*		
Sample no.	3’ G>A %	5’ C>T %	Average damage in %	3’ G>A %	5’ C>T %	Average damage in %
318	0	4	2	17	12	15
320	3	3	3	8	17	13
321	3	2	3	0	0	0
323	1	1	1	20	15	18
324	2	1	1	15	11	13
327	6	9	8	14	9	12
328	8	8	8	11	6	9
329	3	3	3	14	13	14
332	3	4	4	11	15	13
333	6	10	8	11	11	11
334	5	10	7	5	4	5
335	6	10	8	0	0	0
336	5	6	5	5	4	5
