# Supplementary material for: Parasitic infections and resource economy of Danish Iron Age settlement through ancient DNA sequencing
Source: PLoS One. 2018 Jun 20;13(6):e0197399. doi: 10.1371/journal.pone.0197399 (PMC6010210; doi:10.1371/journal.pone.0197399)
Supplement: S9 Table — Amount of phytoliths, diatoms and ash pseudomorphs in sediment samples collected from the central part (at 6 m from the Eastern edge) of the artificial dam in Hoby and their calculated concentrations (per g). Sample weight indicates the portion of the homogenized sediment used for the extraction procedures. The sample is mixed with 50 μl of 6N HCl and 450 μl SPT (3Na2WO4·9WO3·H2O), and a representative aliquot of 50 μl of the supernatant is removed and placed on a microscope slide for counting. Phytoliths and diatoms are counted in 10 evenly distributed fields on a microscope slide at 200x magnification, while ash pseudomorphs were counted in 15 fiels at 400x magnification. The area covered by each field depends on the magnification. 50 μl of the supernatant represent 10% of the total solution. Based on these numbers it is possible to calculate the concentration of phytoliths, diatoms and ash pseudomorphs in each sample. (PDF) [file pone.0197399.s009.pdf]

Sample no.	Area of one field in X200 (or X400) magnification	Weight of sediment sample (mg)	Nos counted	No. of fields counted	phyto./ diatoms /ash p. On slide	phyto. /diatoms /ash p. In 500 µl solution	Phyto. / diatoms / ash p. In 1g sediment
Phytoliths (counted at x200)							
#329	0,785	60	535	10	39256	392561	6542675
#330	0,785	61	536	10	39329	393294	6447447
#331	0,785	57	868	10	63690	636902	11173718
#332	0,785	57	770	10	56499	564994	9912169
#333	0,785	55	636	10	46667	466670	8484910
#334	0,785	56	377	10	27663	276627	4939763
#335	0,785	58	254	10	18637	186375	3213354
#336	0,785	58	125	10	9172	91720	1581375
Diatoms (counted at x200)							
#329	0,785	60	17	10	1247	12474	207898
#330	0,785	61	15	10	1101	11006	180432
#331	0,785	57	162	10	11887	118869	2085417
#332	0,785	57	101	10	7411	74110	1300168
#333	0,785	55	95	10	6971	69707	1267400
#334	0,785	56	39	10	2862	28617	511010

#335	0,785	58	39	10	2862	28617	493389
#336	0,785	58	7	10	514	5136	88557
Ash pseudomorphs (counted at x400)							
#329	0,8821	44	729	15	31735	317352	7212540
#330	0,8821	42	584	15	25423	254230	6053088
#331	0,8821	44	448	15	19503	195026	4432398
#332	0,8821	47	675	15	29384	293844	6252005
#333	0,8821	41	1481	15	64472	644716	15724781
#334	0,8821	48	381	15	16586	165859	3455391
#335	0,8821	49	457	15	19894	198943	4060070
#336	0,8821	45	272	15	11841	118408	2631297
