# Supplementary figures and images for: Parasitic infections and resource economy of Danish Iron Age settlement through ancient DNA sequencing
Source: PLoS One. 2018 Jun 20;13(6):e0197399. doi: 10.1371/journal.pone.0197399 (PMC6010210; doi:10.1371/journal.pone.0197399)

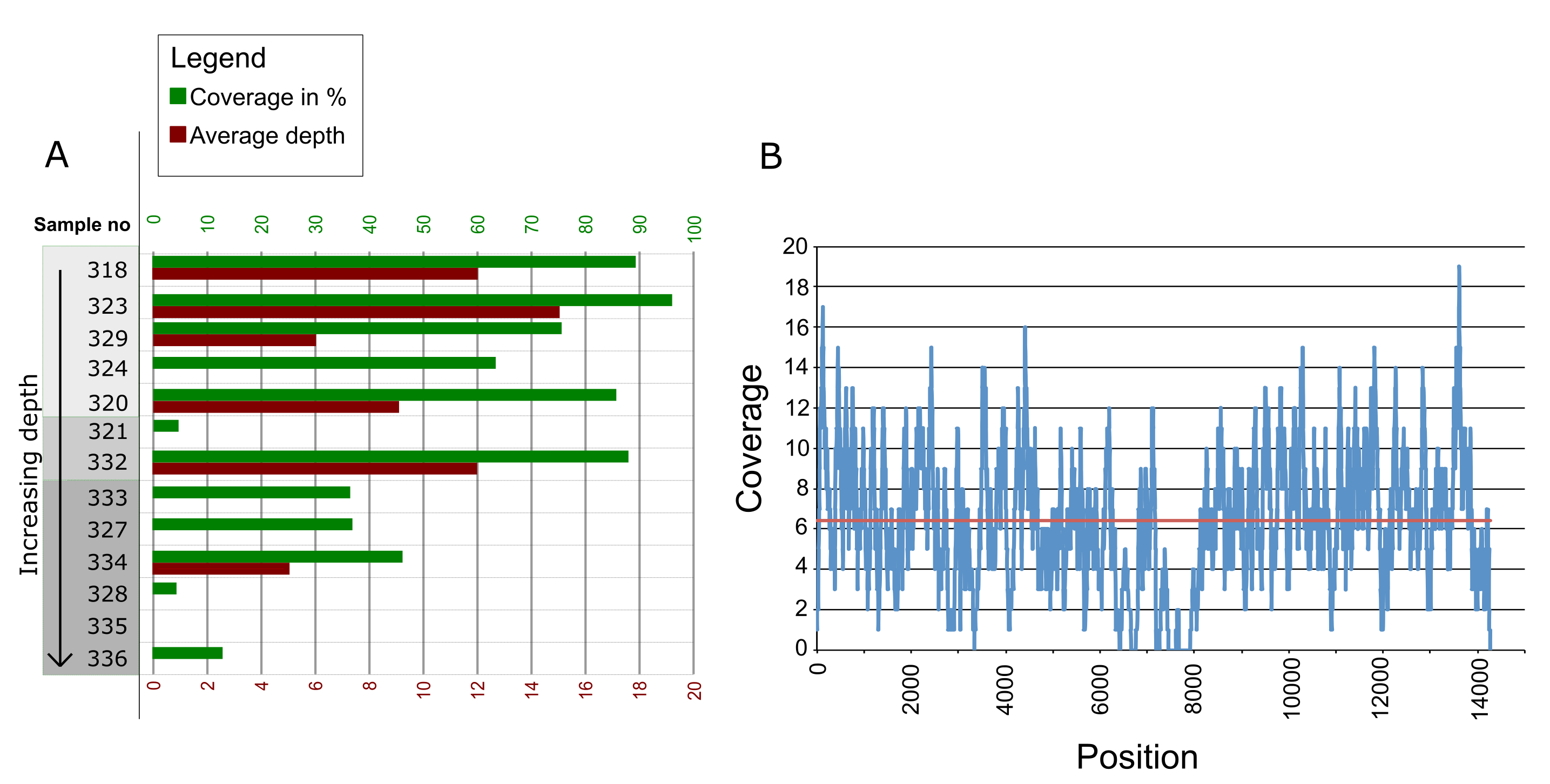

Supplement: S1 Fig — A) Coverage in percent of the mitochondrial genome reference sequence (KC839986) is shown (green) as well as the average depth of coverage (red). Samples from the top, middle and bottom horizons are shaded in light grey, grey and dark grey, respectively. B) Read coverage (blue) for Ascaris spp. reads from sediment sample 323 across the mitochondrial genome, red line shows average coverage (6.42x). (TIF) [file pone.0197399.s010.tif]

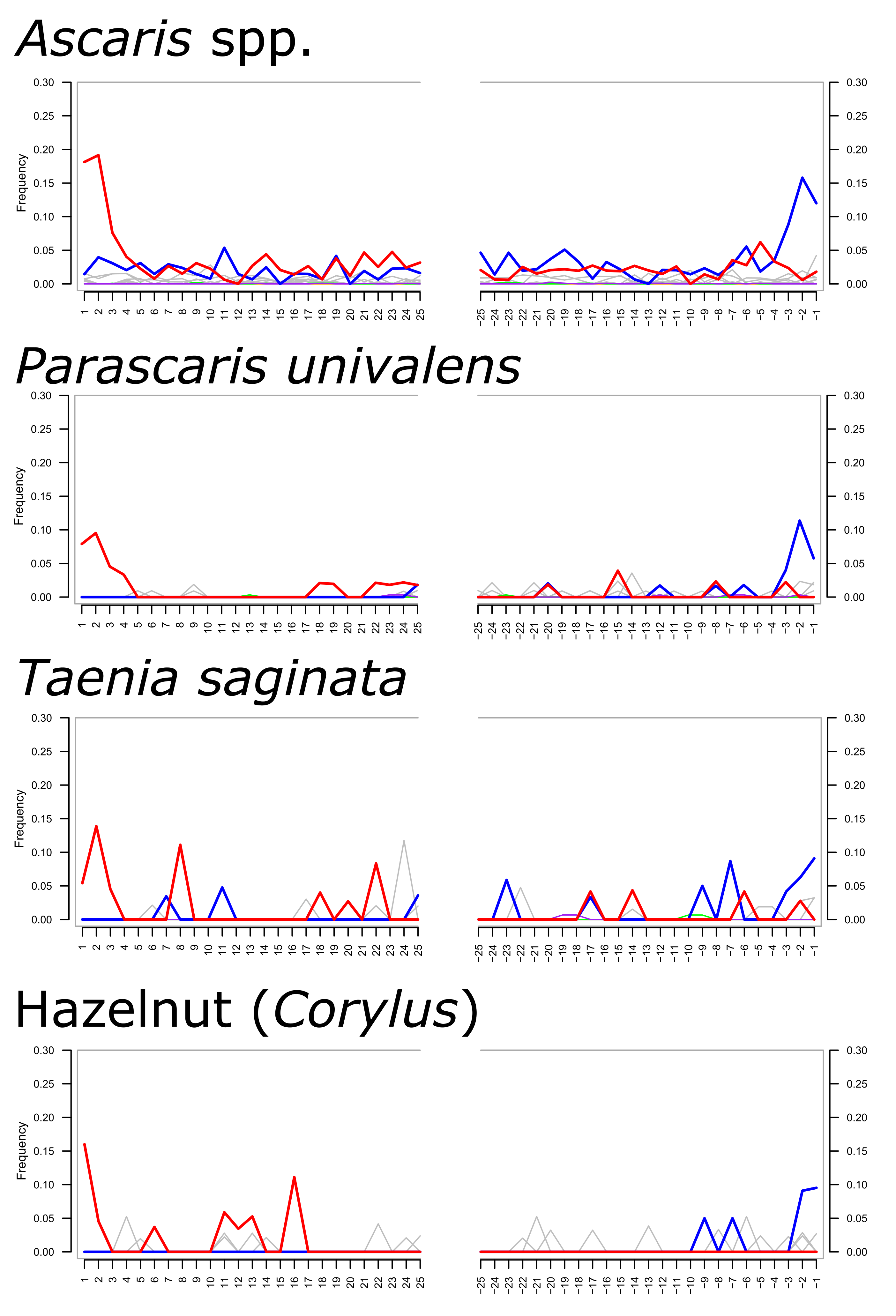

Supplement: S2 Fig — Shows ancient DNA damage profile plots of Ascaris spp. (sample 323, C to T (19%) and G to A (12%))Parascis univalens (sample 334, C to T (8%) and G to A (6%)), Taenia saginata (sample 334, C to T (5%) and G to A (9%)) and hazelnut (Corylus) (sample 327, C to T (16%) and G to A (10%)). C to T (red) and G to A (blue) transitions substitutions for 5’ end of reads (1 to 25 on x-axis) and for 3’ end of reads (-25 to -1 on the x-axis). (TIF) [file pone.0197399.s011.tif]

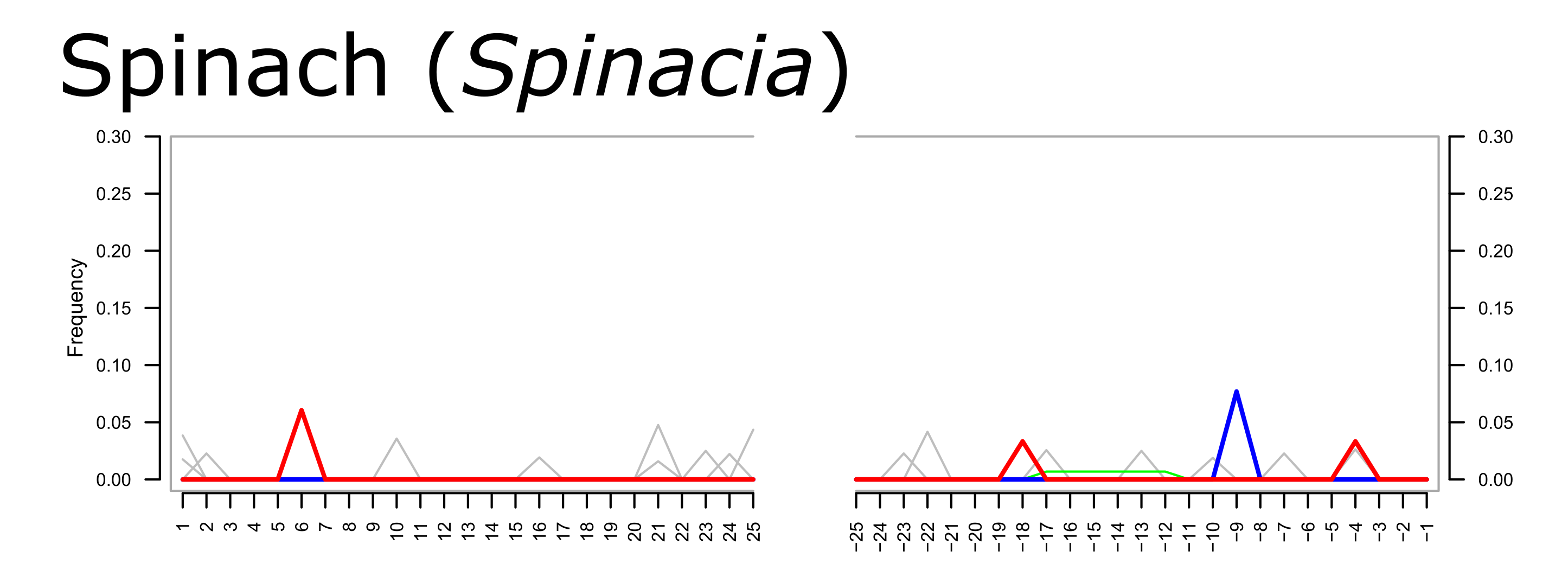

Supplement: S3 Fig — Shows modern DNA damage profile plots of Spinach (Spinacia, sample 329) with no increased damage towards the ends of the reads. C to T (red) and G to A (blue) transitions substitutions for 5’ end of reads (1 to 25 on x-axis) and for 3’ end of reads (-25 to -1 on the x-axis). (TIF) [file pone.0197399.s012.tif]

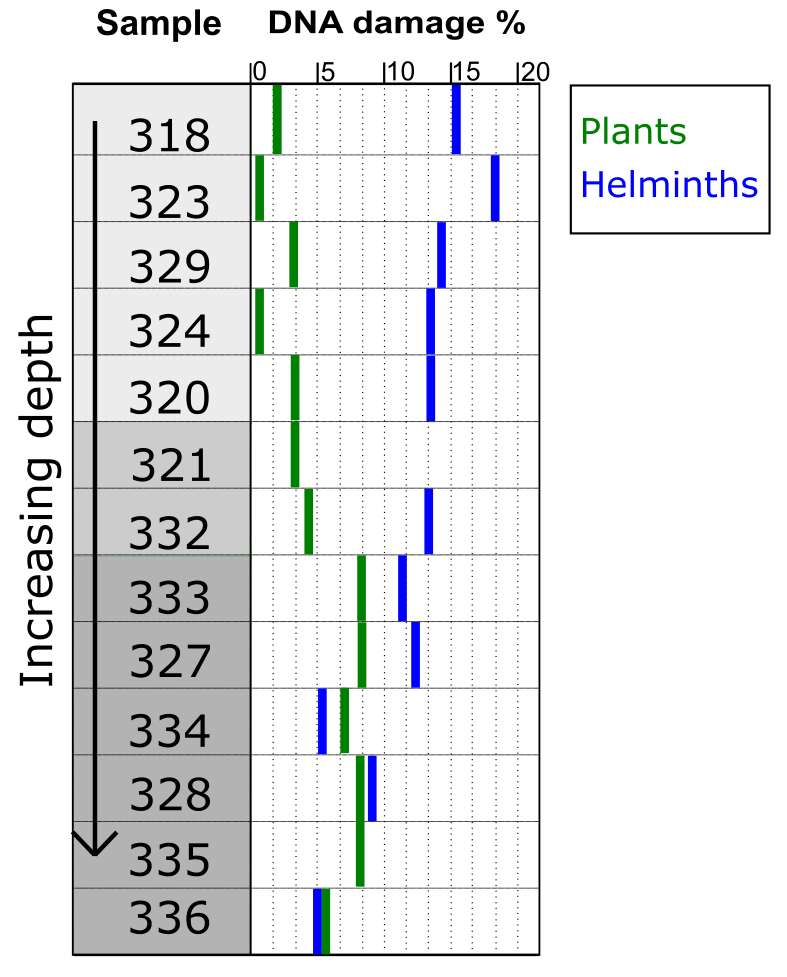

Supplement: S4 Fig — The figure shows the collective ancient DNA damage (C to T and G to A transitions in the 5’and 3’respective ends of assigned reads) as percentage of the 13 samples listed by increasing depth with the top layer (light grey) middle layer (grey) and the bottom layer (dark grey). (TIF) [file pone.0197399.s013.tif]
