# Supplementary material for: Parasitic infections and resource economy of Danish Iron Age settlement through ancient DNA sequencing
Source: PLoS One. 2018 Jun 20;13(6):e0197399. doi: 10.1371/journal.pone.0197399 (PMC6010210; doi:10.1371/journal.pone.0197399)
Supplement: S1 File — (DOCX) [file pone.0197399.s014.docx]

Supplementary Information

Parasitic infections and resource economy of Danish Iron Age settlement through ancient DNA sequencing

Katrine Wegener Tams*, Martin Jensen Søe, Inga Merkyte, Frederik Valeur Seersholm, Peter Steen Henriksen, Susanne Klingenberg, Eske Willerslev, Kurt H. Kjær, Anders Johannes Hansen, Christian Moliin Outzen Kapel*

## Extraction & counting of phytoliths, diatoms, sponges, radiolarians and charred organic material

### Method

Source:

Katz, O; Cabanes, D; Weiner, S; Maeir, AM; Boaretto, E; Shahack-Gross, R (2010). Rapid phytolith extraction for analysis of phytolith concentrations and assemblages during an excavation: an application at Tell es-Safi/Gath, Israel.  [*Journal Of Archaeological Science.*2010, 37:1557-1563.](http://dx.doi.org/10.1016/j.jas.2010.01.016)

Also special acknowledgement to Prof. Stephen Weiner for personal training at Kimmel Center for Archaeological Science

A sediment sample is sieved in order to remove the fraction larger than 0.5 mm. An accurately weighed amount of around 50 mg of this homogenized sediment is placed in a 0.5 ml

conical plastic centrifuge tube. Fifty microliters 6 N HCl are added using an adjustable pipette in order to dissolve carbonate minerals and carbonated hydroxylapatite, including small bone fragments. After the bubbling has ceased (usually a few minutes) 450 ml 2.4 g/ml sodium polytungstate solution (SPT, Na6(H2W12O40)$HO) (Sometu Ltd., Berlin) is added. The tube is vortexed for about 3 s, sonicated for ca.10 min, vortexed again and centrifuged for 10 min at 5000 rpm. At a ﬁnal density of 2.26 g/ml SPT, most of the remaining minerals (mainly quartz and clay) pellet after centrifugation and the phytoliths and other siliceous materials such as diatoms, sponges and radiolarians and charred organic material remain in suspension. Once the phytoliths and other light materials are in, the supernatant is removed to a new 0.5 ml centrifuge tube and vortexed. An aliquot of 50 μl of the supernatant is removed and placed on a microscope slide and covered with a 24 mm cover-slip. Fifty microliters is the maximum volume that allows the solution to completely ﬁll the space between the slide and the cover-slip. The transfer of the 50 μl should be carried out immediately after the vortexing to eliminate any bias due to fractionation within the tube that occurs as the phytoliths, etc. slowly ﬂoat to the top.

The phytolith, etc. concentration can be calculated since the total amount of phytoliths on the slide represents 10% (50 ml out of 500 ml) of the total amount of phytoliths in the initially weighed sample. Note that by transferring the phytoliths in suspension, their distribution on the slide is relatively homogeneous. In previous procedures, the phytoliths are transferred as a dry powder and then mixed with glue (usually Canada Balsam or Entellan). The phytolith distribution on the slide is then relatively heterogeneous and thus necessitates counting the whole slide or a major part of the slide. This is very time consuming.

A general approach we use is to count 10 ﬁelds at 200x magniﬁcation (for phytoliths and diatoms, etc.) or 15 fields at 400x magnification (for ash pseudomorphs and spherulites). (When working on phytolith morphologies we use the standard 400x magniﬁcation in order to reveal detailed morphological characteristics.) For the counting, we randomly choose two ﬁelds around the center of the slide and 8 ﬁelds closer to the periphery. If the variability of these 10 values is high, then additional sets of ten ﬁelds can be counted in the same way, until an acceptable variability is obtained. The slide can also be used for analyzing the assemblage of morphotypes.

As the pH of the extraction solution is very low and the SPT crystallizes within hours once placed on the slide, the remaining supernatant can be diluted and then centrifuged (10 min at 5000 rpm). This pellets the phytoliths. The supernatant is then discarded and the pellet is re-suspended in water, centrifuged and the supernatant is removed again. This is repeated twice in order to remove all traces of the extraction solution. The sample is then air dried in the tube and can be used later for morphotype analysis.

### Theory

Diatoms are the most common component of all aquatic ecosystems and are often being used to assess short- and long-term environmental change. All species are good indicators for a range of water quality variables, because they have narrow optima and tolerances for many environmental variables: habitat, salinity, pH of the water, nutrient, temperature, saprophity, etc. Diatoms respond quickly to environmental change as they immigrate and replicate rapidly and therefore are considered as significant ecological indicators.

Pennate diatoms (encountered at Hoby, min. 8 species in H3 sample) dominate the freshwater, soil and epiphytic niches. Centric diatoms thrive as plankton in marine waters.

In the Hoby case the counting of diatoms is being used to understand the dynamics of the formation and eutrophication of the pond, including seasonality.
